# Taste and chemical composition as drives for utilitarian redundancy and equivalence: a case study in local medical systems in Northeastern Brazil

**DOI:** 10.1186/s13002-022-00503-1

**Published:** 2022-01-25

**Authors:** Rafael Corrêa Prota dos Santos Reinaldo, Flávia Rosa Santoro, Ulysses Paulino Albuquerque, Patrícia Muniz de Medeiros

**Affiliations:** 1 Laboratório de Ecologia e Evolução de Sistemas Socioecológicos, Departamento de Botânica, Centro de Biociências, Universidade Federal de Pernambuco, Cidade Universitária, Recife, PE 50670-901 Brazil; 2 Programa de Pós-Graduação em Botânica, Universidade Federal Rural de Pernambuco, Rua Dom Manuel de Medeiros s/n, Dois Irmãos, Recife, Pernambuco 52171-900 Brazil; 3 Instituto Multidisciplinario de Biologia Vegetal (IMBIV), CONICET – Universidad Nacional de Córdoba, Avenida Vélez Sársfield 299, Córdoba, Argentina; 4 Centro de Ciências Agrárias, Universidade Federal de Alagoas, Rio Largo, Alagoas Brazil

**Keywords:** Ethnobotany, Organoleptic properties, Selection criteria, Traditional knowledge, Use patterns

## Abstract

**Background:**

We aimed to verify whether the taste and chemical composition influence the selection of plants in each medicinal category, whether within a socio-ecological system or between different socio-ecological systems. To this end, we use the theoretical bases of the Utilitarian Redundancy Model and the Utilitarian Equivalence Model. We studied the local medical systems of four rural communities in northeastern Brazil, used as models to test our assumptions.

**Methods:**

The data on medicinal plants and local therapeutic function were obtained from semi-structured interviews associated with the free-listing method, allowing to generate indexes of similarity of therapeutic use between the plants cited in each region. During the interviews, each informer was also asked to report the tastes of the plants cited. Subsequently, we classified each plant in each region according to the most cited taste. The data about the chemical composition of each plant were obtained from a systematic review, using Web of Knowledge and Scopus databases.

**Results:**

Pairs of plants with similar tastes are 1.46 times more likely to have the same therapeutic function within a local medical system (redundancy), but not between medical systems (equivalence). We also find that chemical compounds are not primarily responsible for utilitarian redundancy and equivalence. However, there was a tendency for alkaloids to be doubly present with greater expressiveness in pairs of equivalent plants.

**Conclusions:**

The results indicate that each social group can create its means of using the organoleptic characteristics as clues to select new species as medicinal. Furthermore, this study corroborates the main prediction of the Utilitarian Equivalence Model, that people in different environments choose plants with traits in common for the same functions.

## Background

The production of knowledge that constitutes local medical systems is influenced by several factors associated with human perception about the natural resources, such as its perceived availability and organoleptic properties, but also the intrinsic characteristics of the resources used, such as their chemical compounds [1, 2]. Evidence in the literature shows that people from different regions of the world have similar patterns of use of medicinal plants [3], which may mean that these factors exert the same influence on different human populations, being responsible for the selection of medicinal species.

Among the organoleptic properties of medicinal plants, the taste has been mentioned in several studies as a clue to the inclusion of medicinal plants in local medical systems [4–14]. In general, these studies suggest that local populations use plant taste attributes to distinguish between medicinal and nonmedicinal plants, but few studies find a clear pattern on which tastes are attributed to which therapeutic functions. For example, in Patagonia Argentina, Morales and Ladio [14] reported that stomach problems are usually treated with sweet plants. On the other hand, in a municipality belonging to Chiapas, Mexico, Brett [5] found that the local population preferred plants with a sweet-smelling flavor to treat diseases related to the respiratory system, while the treatment of poisonous animals’ bites was mostly conducted with the use of bitter plants. In other locations, bitter plants are used to treat gastrointestinal disorders such as diarrhea and dysentery [12, 13].

The chemical compounds may explain the importance of taste in selecting medicinal plants [15, 16]. Terpenoids, flavonoids, tannins, and other chemical components that have pharmacological activity are usually related to the flavors presented in plants [7, 12]. Nonetheless, different chemical compounds present the same taste [9]—mostly the bitter taste—and taste alone may not fully explain the selection of plants for different therapeutic functions. It is possible that chemical compounds are also responsible for the selection of medicinal plants by local populations, albeit indirectly—for example through the perception of effectiveness, as suggested by Medeiros et al*.* [1].

In this study, we seek to understand whether there is a pattern that relates taste and/or chemical compounds to specific medicinal functions within and between local medical systems. As mentioned, there are similar patterns of phylogenetic-based plant selection in different environments [3] (see also Reinaldo et al*.* [17]), but patterns that relate flavors and/or chemical composition to the same medicinal functions in different medical systems have not yet been seen.

If the taste and/or chemical composition of a plant influences in the same way the selection of medicinal plants by human groups, we would expect plants with the same taste and/or similar chemical composition to have the same medicinal function within and between cultural groups. In this sense, we can use two models that analyze the functions of medical systems: The Utilitarian Redundancy Model (URM) and the Utilitarian Equivalence Model (UEM) (Box 1). Those models allow us to identify the influence of taste and chemical compounds in the selection of plants to each medical function within a medical system (URM) and between distinct medical systems, formed by people living in distinct cultural and environmental contexts (UEM).

**Box 1:** The utilitarian redundancy model and the utilitarian equivalence model

**Utilitarian redundancy model** (URM): the URM derives from the ecological redundancy [18] by adopting a functional perspective in the analysis of natural resource use by human populations, and it evaluates the role of functional overlap within a socioecological system (see Albuquerque and Oliveira [19]). This analytical perspective arose from the observation that several species within a local medical system are used for the same therapeutic function—for example, mint and lemon can be both used to treat colds—they are culturally redundant regarding local use indications [20]. The model can be applied to any function of a socio-ecological system, not only medical ones. The URM model is based on the following assumptions: (a) species have different functions within social-ecological systems, but a level of overlap in function (i.e., redundancy) occurs; (b) increased redundancy promotes resilience in social-ecological systems, and (c) redundancy depends on the knowledge characteristics and practices of a given human community [20]. For this study, we propose that redundant species in a medical system were selected for the same therapeutic function because they have a similar taste or chemical components	
**Utilitarian equivalence model** (UEM): The UEM is an operational concept based on Odum's [21] ecological equivalence model, which aims to understand the cases of overlapping of useful species in different socio-ecological systems [17]. Utilitarian equivalence, thus, indicates species that are used for the same purposes or similar purposes (not only medicinal ones) in different socioecological systems. Especially for medicinal use, equivalent species provides the ideal scenario to seek common selection criteria, in order to identify the shared characteristics among the equivalent pairs and consequently the main types of perceptions or stimuli, which led to the inclusion of such species in different local medical systems [17]. The model assumes that: (a) utilitarian equivalence, understood as the high overlap of use between two species in distinct socio-ecological systems, is relative and not absolute, since, in the absence of intrinsically identical plant species or culturally equal peoples, the medicinal uses are not necessarily identical, but rather similar; (b) equivalence is due to two groups of complementary variables, cultural traits and environmental factors; (c) the evolutionary events that led to utilitarian equivalence may be associated with the similarity between intrinsic characteristics of useful species. Thus, in this study we rely on one of the predictions of the UEM, that plants of distinct medical systems tend to share certain traits in common, such as taste and chemical compounds	

Following this rationale, we propose two hypotheses that try to understand the role of taste and chemical compounds on the selection of plants with therapeutic functions:

1. Species with similar tastes tend to present Utilitarian Redundancy and Utilitarian Equivalence. It is expected that, within a socio-ecological system, pairs of species that have similar tastes are more likely to have the same therapeutic function (redundancy) than pairs of species with different tastes. We also expect that, in distinct socio-ecological systems, pairs of species that have similar tastes are more likely to have the same therapeutic function (equivalence) than plants with different tastes.
2. Species with similar chemical compounds tend to present Utilitarian Redundancy and Utilitarian Equivalence. It is expected that, within a socio-ecological system, pairs of species that have chemical compounds of the same class are more likely to have the same therapeutic function (redundancy) than pairs of species with chemical compounds of different classes. We also expect that, in distinct socio-ecological systems, pairs of species that have chemical compounds of the same class are more likely to have the same therapeutic function (equivalence) than plants with chemical compounds of different classes.

## Methods

### Study area

The study was conducted in four rural communities located in the Northeast of Brazil, two of them included in the Catimbau National Park (S 08° 7′ 23′′ W 37° 09′ 21′′), a semi-arid region in the state of Pernambuco, and the other two included in the Murici Ecological Station (S 9° 18′ 26′ ′W 35° 55′ 55′′), a hot and humid climate region in the State of Alagoas. Communities were chosen based on their proximity and dependence on natural resources. In addition, we selected two regions of climate and water availability that were deeply different and with potentially different local floras because we intended to evaluate if under different socio-ecological conditional there is the same pattern of plant selection.

The Catimbau National Park has xerophytic vegetation locally known as caatinga. It is classified as a Seasonally Dry Tropical Forest (SDTF), with many deciduous, thorny, and succulent species. The cultural formation of local populations is marked by a strong influence of the Catholic and Protestant Christian religions. The communities studied were Igrejinha and Batinga, which are located in the region of the park belonging to the municipality of Buíque/PE, distant about 20 km from the urban area. The local climate is Bsh type, according to Köppen's classification [22] with rainfall regimes ranging from 650 to 1100 mm, usually restricted from October to January. The community of Igrejinha has a population of 171 inhabitants, while Batinga has 71 inhabitants. The medical service is offered by the municipality only in Vila do Catimbau, about ten kilometers from the communities. In addition, a Non-Governmental Organization (NGO) called Amigos do Bem (“Friends of the good”) offers medical care and distribution of medicine to families in social vulnerability. Despite this, both communities have local plant-based medical systems, and the medicinal use of these resources is mainly made of teas and infusions.

The Murici Ecological Station, included in the municipality of Murici/AL, is covered by the Atlantic Rainforest. The Atlantic Rainforest, classified as Tropical Rainforest (TR), is considered one of the world's hotspots, with about 20 thousand species of vascular plants, of which 40% are endemic [23]. Its tropical hot-humid climate, type AS according to the classification of Koppen, is marked by annual rainfall ranging from 800 to 1800 mm and annual temperatures around 25 °C [24]. The selected local communities were Settlement Che Guevara and Settlement Dom Helder Câmara, distancing about 13 km from the urban area. Both communities have as main activity family farming. A total of 204 people live in the settlement Dom Hélder Câmara, while the Settlement Che Guevara has 220 residents. Among the residents, prevail the Christian religious doctrines. The two communities have medical systems that are highly dependent on the use of plants, with a clear preference for these resources over biomedicine, for example. In both communities, there are common practices of magical-religious rituals from folk healers. Hospitals and health centers are restricted to the urban area, about 13 km away from the communities, and are often reached thanks to school buses from the municipality.

### Ethnobotanical survey

Most of the data were obtained from semi-structured interviews associated with the free-listing method and carried out with local specialists in medicinal plants, providing greater reliability of data on the repertoire of medicinal plants. This survey took place between January 2017 and January 2018 with monthly visits. We applied the snowball technique for the selection of experts [25]. In this technique, a first informant is asked to refer people in the community who have a piece of rich knowledge about the subject. Then, the referred ones also refer to other informants. Only people over the age of 18 were included in the survey. In total, 127 people were interviewed, 49 from the TR areas and 88 from the SDTF area (see Table 1). In the interviews, the subjects were submitted to the following questions: (1) What medicinal plants do you know?—for this question, we use the free list technique, encouraging participants to cite the maximum number of known medicinal plants; (2) What is the use of each of these plants?—with this question we access the medicinal function of the mentioned plants; (3) Does this plant have any taste? Which?—with this question, we access the taste of the mentioned plant as perceived by the participants.

**Table 1:** Distribution of the interviewed local experts by community and by gender (sex)

Ecosystem	Community	Men	Women	Total
TR	Ass. D. Helder Câm	15	11	26
	Ass. Che Guevara	11	2	13
SDTF	Igrejinha	26	34	59
	Batinga	13	16	29
		65	63	127

### Collection of botanical material and taxonomic identification

The medicinal plants mentioned were collected in the region with the assistance of local experts. The identification of the species and the register of the testimonial material were carried out by the Instituto Agronômico de Pernambuco (IPA).

Regarding the collection of the above-mentioned plants, a request to carry out scientific research in the areas of the Catimbau National Park (PARNA Catimbau) and the Murici Ecological Station was made to ICMBio/SISBIO, an agency of the Brazilian Ministry of the Environment (MMA).

### Treatment of interview data

The information collected from the informants was filtered by the inclusion and exclusion criteria described below.

#### Preliminary screening of diseases and plant species: inclusion and exclusion criteria

We considered only the diseases mentioned by two or more informants from a given region. Thus, if plant 'A' of the SDTF was only referred to 'influenza' and 'gastritis' uses and these data were mentioned by a single informant, those data were ignored, and consequently, plant A was excluded from the analyses. Likewise, if in a given region none of the data on how to cure a particular disease was shared by two or more people, the disease was excluded from the analyses. This process aimed to minimize the chances that idiosyncratic information would bias the findings.

#### Further screening of diseases and plant species: inclusion and exclusion criteria

The current study falls into the etic approach, characterized by analyzing the phenomena from the researcher's perspective. Within this proposal, we consider only diseases and symptoms recognized by biomedicine. Some diseases considered in this study have a certain level of overlap with each other, such as diseases related to the respiratory system. However, in the local medical system, informants indicated that these were different things. In the case of expectorants, it was clear that they hoped the plant would help the body "throw it out." In contrast, when informants used plants to treat cough, the informants were aware that the goal was to stop the "spasms," which in turn would make expectorant action difficult. Following this distinction between citations of diseases and symptoms that fit or not for medical use from a biomedical viewpoint, only the diseases that occurred in both SDTF and TR were selected. There was also the exclusion of some species that treated only isolated diseases, occurring in only one of the regions.

### Obtaining the phytochemical data of the cited plants

#### Literature research strategy

The database with the phytochemical profile of the plants included in the study comprised information on the presence or absence of the main classes of compounds, namely tannins, terpenes, alkaloids, flavonoids, and phenols. Data collection was based on information found in the available literature and recent research in the area. Pubmed, Scopus, and Web of Knowledge were consulted. The keywords used included the name of the respective species, "Species name + Ethnopharmacology," "Species name + chemical," "Species name + bioprospecting" and "specific name + Pharmacology." For each species, only studies analyzing at least one of the useful plant parts were considered.

We obtained 90 studies with information on 55 of the 64 selected medicinal species. However, to avoid bias, only studies that analyzed parts of the plants used by informants were considered. The papers found are shown in Table 2, and the phytochemical data extracted from them are included in Table 3.

**Table 2:** List with the medicinal species considered in the study and the papers consulted

Plant species	References
*Acanthospermum hispidum* DC.	Chika et al. [26]; Sanon et al. [27]; N'do et al. [28]; Tirloni et al. [29]; Ganfon et al. [30]; Arena et al. [31]; Roy et al. [32]
*Aloe vera* (L.) Burm*.* f.	Benzidia et al.[33]; Kumar et al.[34]; Cock [35]; Guo and Mei [36]; Salehi et al. [37]; Taukoorah and Mahomoodally [38]; Mariita et al.[39]
*Amburana Cearensis* (Allemão) A.C.Sm.	Costa et al. [40]; Canuto and Silveira [41]; Leal et al. [42]; Costa-Lotufo et al. [40]; Farias et al. [43]
*Anacardium occidentale* L.	Mustapha et al. [44]; Tedong et al. [45]; Carvalho et al. [46]; Souza et al. [47]
*Anadenanthera colubrina* var. cebil (Griseb.) Altschul	Lima Neto et al. [48]; Vigerelli et al. [49]; Cartaxo et al. [50]; Gutierrez-Lugo et al. [51]; Melo et al. [52]; Damascena et al. [53]
*Astraea lobata* (L.) Klotzsch	Ezeabara and Okonkwo [54]
*Bauhinia acuruana* Moric.	Trentin et al. [55]; Gois et al. [56]
*Borreria verticillata* (L.) G. Mey.	Ushie et al. [57]; Baldé et al. [58]; Moreira et al. [59]
*Commiphora leptophloeos* (Mart.) J.B.Gillett	Clementino et al. [60]
*Copaifera lucens* Dwyer	Santos et al. [61]
*Cymbopogon citratus* (DC) Stapf.	Geetha and Geetha[62]; Shah et al. [63]; Cheel et al. [64]
*Dysphania ambrosioides* (L.) Mosyakin and Clemants	Loufoua et al. [65]; Zohra et al. [66]; Soares et al. [67]; Mwanauta et al. [68]
*Genipa americana* L.	Barbosa et al. [69]; Soares et al. [67]
*Handroantus impetiginosus* (Mart.ex DC.) Mattos	Pires et al. [70]
*Hymenaea courbaril* L.	Bezerra et al. [71]; Cecílio et al. [72]; Aleixo et al. [73]
*Jatropha gossypiifolia* L.	Félix-Silva et al. [74]
*Libidibia ferrea* (Mart. Ex Tul.) L.P.Queiroz	Comandolli-Wyrepkowski et al. [75]
*Lippia origanoides* Kunth	Pinto et al. [76]
*Mentha piperita* L.	Ramkissoon et al. [77]; Zheljazkov and Astatkie [78]
*Mimosa tenuiflora* (Willd.) Poir.	Bezerra et al. [71]
*Myracrodruon urundeuva* Allemão	Cecílio et al. [72]
*Ocimum gratissimum* L.	Kpadonou-Kpoviessi et al. [79]; Aba and Udechukwu [80]
*Passiflora cincinnata* Mast.	Siebra et al. [81]
*Passiflora foetida* L.	Patil and Paikrao [82]
*Phyllanthus urinaria* L.	Xu et al. [83]; Chung et al. [84]
*Plinia cauliflora (Mart.)* Kausel	Chavasco et al. [85]; Oliveira et al. [86]
*Psidium guajava* L.	Cecílio et al. [72]; Morais-Braga et al. [87]
*Psidium guineense* Sw.	González et al. [88]
*Punica granatum* L.	Rajeswari [89]; Calín-Sánchez et al. [90]
*Rosmarinus officinalis* L.	Btissam et al. [91]; Pérez‐Mendoza et al. [92]
*Ruta graveolens* L.	Amabye and Shalkh [93]; Sampaio et al. [94]
*Sambucus nigra* L.	Akhtar et al. [95]
*Schinopsis brasiliensis* Engl.	Lima-Saraiva [96]; Barbosa et al. [69]
*Schinus terebinthifolia* var. acutifolia Engl.	Abdul-Hafeez et al. [97]
*Solanum paniculatum* L.	Lôbo et al. [98]
*Syzygium cumini* (L.) Skeels	Akhtar et al. [95]; Tripathi and Kohli [99]
*Vismia guianensis* (Aubl.) Choisy	Camelo et al. [100]
*Ximenia americana* linn	Gaichu et al. [101]; Aragão et al. [102]
*Ziziphus joazeiro* Mart.	Brito et al. [103]; Andrade et al. [104]

**Table 3:** List with the plant species included in the chemical analyzes and their respective therapeutic indications

Species	Family	Common name	Origin	Classes of compounds	Region	Part of the plant used	Taste	Therapeutic indications	Herbarium voucher
*Acanthospermum hispidum* DC	Asteraceae	Federação	Native	Alk, Phe, Fla, Tan and Ter	SDTF	Leaves and roots	Tasteless	Expectorant, cough, flu and colds	IPA91626
					TR	Flower and roots	Tasteless	Cough	Sterile material
*Aloe vera* (L.) Burm. f	Asphodelaceae	Babosa	Exotic	Alk, Phe, Fla, Tan and Ter	SDTF	Bark, leaves and roots	Bitter	Expectorant, injury, stomach problems, flu and colds, cough and worms	Sterile material
*Amburana Cearensis* (Allemão) A.C.Sm	Fabaceae	Imburana de cheiro	Native	Alk, Phe, Fla, Tan and Ter	SDTF	Bark, leaves and seeds	Bitter	Diarrhea, Headache, Flu and colds, Indigestion and Cough	Sterile material
*Anacardium occidentale* L	Anacardiaceae	Cajueiro roxo	Native	Alk, Phe, Fla, Tan and Ter	SDTF	Bark and leaves	Astringent	Toothache, Injury, Stomach problems and Inflammation in general	Sterile material
					TR	Bark and fruit	Astringent	Toothache, Injury, Gynecological and Problem	Sterile material
*Anadenanthera colubrina* var. cebil (Griseb.) Altschul	Fabaceae	Angico	Native	Phe, Fla, Ter and Tan	SDTF	Bark	Bitter	Injury, Flu and colds and Inflammation in general	IPA91649
*Astraea lobata* (L.) Klotzsch	Euphorbiaceae	Alfavaca de cobra	Native	Alk, Phe, Fla, Tan and Ter	TR	Roots	Tasteless	Stinging of venomous animals	Sterile material
*Bauhinia acuruana* Moric	Fabaceae	Mororó	Native	Phe and Ter	SDTF	Bark, leaves and roots	Bitter	Diabetes, Flu and colds, Inflammation in general and Cough	IPA91660
*Borreria verticillata* (L.) G. Mey	Rubiaceae	Vassoura de botão	Native	Alkaloid, flavonoid Terpene	TR	Entire plant or roots	Bitter	Stroke	IPA91713
*Commiphora leptophloeos* (Mart.) J.B.Gillett	Burseraceae	Imburana de cambão	Native	Alk, Phe, Fla, Tan and Ter	SDTF	Bark and leaves	Astringent	Diarrhea, Injury, Hypertension and Cough	IPA91663
*Copaifera lucens* Dwyer	Fabaceae	Pau D’óleo	Native	Ter	TR	Sap and resin	Bitter	Pain in general and stroke	Sterile material
*Cymbopogon citratus* (DC) Stapf	Poaceae	Capim Santo	Exotic	Phe, Fla, Tan and Ter	SDTF	Leaves	Sweet	Calming, Diarrhea, Flu and colds, Hypertension and Indigestion	Sterile material
					TR	Leaves	Sweet	Diarrhea	Sterile material
*Dysphania ambrosioides* (L.) Mosyakin and Clemants	Amaranthaceae	Mastruz	Exotic	Alk, Phe, Fla, Tan and Ter	SDTF	Entire plant or roots	Bitter	Expectorant, Injury, Bone fracture, Flu and colds, Cough, Worms and Stomach problems	IPA91613
					TR	Leaves	Bitter	Expectorant, Flu and colds, Cough and Worms	IPA91714
*Genipa americana* L	Rubiaceae	Genipapo	Native	Phe, Tan and Ter	TR	Bark and fruit	Sweet	Anemia	Sterile material
*Guazuma ulmifolia* Lam	Malvaceae	Mutamba	Native	NA	TR	Bark	Bitter	Bone fracture and Cough	IPA91718
*Handroantus impetiginosus* (Mart.ex DC.) Mattos	Bignoniaceae	Pau D’arco Roxo	Native	Phe and Fla	SDTF	Bark	Bitter	Stomach problems	Sterile material
					TR	Bark	Bitter	Wounds	Sterile material
*Hymenaea courbaril* L	Fabaceae	Jatobá	Native	Alk, Phe, Fla, Tan and Ter	SDTF	Bark, fruit and roots	Astringent	Anemia, Expectorant, Injury, Stomach problems, Flu and colds, Airway inflammation, Inflammation in general and Cough	IPA91630
					TR	Bark and fruit	Astringent	Injury and Airway inflammation	Sterile material
*Jatropha gossypiifolia* L	Euphorbiaceae	Pinhão Roxo	Native	Alk, Phe, Fla, Tan and, Ter	SDTF	Leaves, shoot, sap and resin	Astringent	Stinging of venomous animals	IPA91702
*Libidibia ferrea* (Mart. ex Tul.) L.P.Queiroz	Fabaceae	Jucá	Native	Phe, Fla and Terpene	SDTF	Bark and fruit	Bitter	Toothache	IPA91696
*Lippia origanoides* Kunth	Lamiaceae	Alecrim do Mato	Native	Phe, Fla and Ter	SDTF	Leaves	Spicy	Toothache and Headache	IPA91612
*Maranta* sp.	Maranthaceae	Uruba	Native	NA	TR	Roots	Tasteless	Stinging of venomous animals	Sterile material
*Mentha piperita* L	Lamiaceae	Hortelã da folha pequena	Exotic	Phe, Fla, Tan and Ter	SDTF	Leaves and roots	Sweet	Expectorant, Flu and colds, Airways Inflammation and Cough	Sterile material
*Mimosa tenuiflora* (Willd.) Poir	Fabaceae	Jurema Preta	Native	Alk, Phe, Fla, Tan and Ter	SDTF	Bark	Bitter	Injury	Sterile material
*Myracrodruon urundeuva* Allemão	Anacardiaceae	Aroeira	Native	Fla, Tan and Ter	SDTF	Bark, stem, flower and leaves	Bitter	Pain in general, Acariasis and other infestations, Injury, Stomach problems, Indigestion, Inflammation in general and Cough	Sterile material
*Neoglaziovia variegata* (Arruda) Mez	Bromeliaceae	Caruá	Native	NA	SDTF	Roots	Sweet	Spine problems	IPA91701
*Ocimum gratissimum* L	Lamiaceae	Alfavaca	Exotic	Alk, Phe, Fla, Tan and Ter	TR	Leaves	Astringent	Stomach problems and conjunctivitis	Sterile material
*Passiflora cincinnata* Mast	Passifloraceae	Maracujá do Mato	Native	Alk, Phe, Fla and Tan	SDTF	Leaves, fruit, roots and seeds	Sour	Calming, Flu and colds, Inflammation in general and Cough	IPA91635
*Passiflora edulis* Sims	Passifloraceae	Maracujá	Native	NA	SDTF	Leaves	NA	Indigestion	Sterile material
*Passiflora foetida* L	Passifloraceae	Maracujá de Estralo	Native	Alk, Phe, Fla and Tan	SDTF	Leaves	Tasteless	Flu and colds and Conjunctivitis	IPA91677
*Periandra mediterranea* (Vell.) Taub	Fabaceae	Alcançu	Native	NA	SDTF	Bark, leaves and roots	Sweet	Expectorant, Flu and colds, Airways inflammation and Cough	IPA91648
*Persea americana* Mill	Lauraceae	Abacate	Exotic	NA	SDTF	Leaves	Tasteless	Renal problems	HST22158
*Phyllanthus urinaria* L	Phyllanthaceae	Quebra Pedra	Native	Phe, Fla Tan and Ter	SDTF	Entire plant and roots	Bitter	Renal problems	Sterile material
					TR	Entire plant or roots	Bitter	Renal problems	Sterile material
*Pityrocarpa moniliformis* (Benth.) Luckow and R.W.Jobson	Fabaceae	Canzenzo	Native	NA	SDTF	Bark	Astringent	Diarrhea	IPA91651
*Plectranthus amboinicus* (Lour.) Spreng	Lamiaceae	Hortelã da folha grande	Exotic	NA	SDTF	Leaves	Sweet	Flu and colds and Cough	Sterile material
*Plinia cauliflora* (Mart.) Kausel	Myrtaceae	Jabuticaba	Native	Alk, Phe, Fla Tan and Ter	SDTF	Bark	Astringent	Diarrhea	Sterile material
*Plumbago scandens* L	Plumbaginaceae	Louco	Native	NA	SDTF	Stem, leaves and roots	Tasteless	Toothache	HST22163
*Poincianella microphylla* (Mart. ex G.Don) L.P.Queiroz	Fabaceae	Catingueira rasteira	Native	NA	SDTF	Bark, flower and roots	Tangy	Inflammation in general and Cough	IPA91653
*Pombalia arenaria* (Ule) Paula-Souza	Violaceae	Papaconha	Native	NA	SDTF	Bark and roots	Tasteless	Expectorant, Flu and colds, Airways Inflammation and Cough	IPA91628
*Prosopis juliflora* (Sw.)	Fabaceae	Algaroba	Exotic	NA	SDTF	Bark	Bitter	Inflammation in general	Sterile material
*Protium heptaphyllum* (Aubl.)	Burseraceae	Amescla	Native	NA	TR	Sap/resin and Seeds	Sweet	Toothache, Stomach problems	Sterile material
*Psidium guajava* L	Myrtaceae	Goiaba	Exotic	Phe, Fla, Tan and, Ter	SDTF	Bark and leaves	Bitter	Diarrhea	Sterile material
*Psidium guineense* Sw	Myrtaceae	Araçá	Native	NA	TR	Leaves	Bitter	Diarrhea	IPA91708
*Punica granatum* L	Punicaceae	Romã	Exotic	NA	SDTF	Bark, leaves, fruit and seeds	Bitter	Throat problems, Stomach problems and Inflammation in general	Sterile material
*Rosmarinus officinalis* L	Lamiaceae	Alecrim	Exotic	Alk, Phe, Fla Tan and Terpene	SDTF	Leaves	Spicy	Headache, Flu and colds	Sterile material
*Ruta graveolens* L	Rutaceae	Arruda	Exotic	Alk, Phe, Fla Tan and Ter	SDTF	Leaves	Bitter	Headache, Pain in general	Sterile material
*Sambucus nigra* L	Adoxaceae	Sabugueira	Exotic	Alk, Phe and TerAlk, Phe, Fla, Tan and Ter	SDTF	Flower	Tasteless	Flu and colds and Cough	HST22162
					TR	Flower and leaves	Bitter	Colds and flu,	Sterile material
*Schinopsis brasiliensis* Engl	Anacardiaceae	Baraúna	Native	NA	SDTF	Bark, stem, leaves, sap and resin	Astringent	Headache, Flu and colds	Sterile material
*Schinus terebinthifolia* var. *acutifolia* Engl	Anacardiaceae	Aroeira	Native	Alk, Phe, Fla Tan and Ter	TR	Bark, leaves	Astringent	Injury, General inflammation and Gynecological problem	Sterile material
*Senegalia bahiensis* (Benth.) Seigler and Ebinger	Fabaceae	Carcará	Native	Alk, Phe, Fla, Tan and Ter	SDTF	Bark and roots	Bitter	Spine problems and Kidney problems	IPA91697
*Senna occidentalis* (L.) Link	Fabaceae	Mangerioba	Native	NA	TR	Flower and seeds	Bitter	Headache, Airways Inflammation	IPA91706
*Senna spectabilis* var. *excelsa* (Schrad.) H.S.Irwin and Barneby	Fabaceae	Canafístula	Native	NA	SDTF	Bark	Bitter	Diarrhea	HST22166
*Sideroxylon obtusifolium* (Roem. and Schult.) T.D.Penn	Sapotaceae	Quixabeira	Native	Alk, Phe, Fla, Tan and Ter	SDTF	Bark	Astringent	Injury, General inflammation, Stroke and Gynecological problem	Sterile material
*Solanum paniculatum* L	Solanaceae	Jurubeba	Native	Alk, Phe, Fla Tan and Ter	SDTF	Leaves, fruit, roots and seeds	Bitter	Injury, Stomach problems, Flu and colds, Inflammation in general and Cough	IPA91633
					TR	Bark, flower, leaves, fruit, entire plant, roots, sap/resin and seeds	Bitter	Expectorant, Flu and colds and Cough	IPA91709
*Sorocea* sp.	Moraceae	Pau Teiu	Native	NA	SDTF	Bark, sap and resin	Bitter	Stinging of venomous animals	Sterile material
*Spondias tuberosa* L	Anacardiaceae	Umbuzeiro	Native	NA	SDTF	Bark and leaves	Sour	Calming, Diarrhea and Insomnia	Sterile material
*Syagrus coronata* (Mart.) Becc	Arecaceae	Coco Ouricuri	Native	NA	SDTF	Roots	Sweet	Airways inflammation and Spine problems	Sterile material
*Syzygium cumini* (L.) Skeels	Myrtaceae	Azeitona Roxa	Exotic	NA	TR	NA	Tasteless	Diabetes	Sterile material
*Tapirira guianensis* Aubl	Anacardiaceae	Cupiuba	Native	NA	TR	Sap and resin	NA	Injury	Sterile material
*Tarenaya spinosa* Jacq.) Raf	capparaceae	Mussambe	Native	Alk, Phe, Fla, Tan and Ter	TR	Flower and roots	Bitter	Flu and colds	Sterile material
*Tocoyena formosa* (Cham. and Schltdl.) K.Schum	Rubiaceae	Genipapo	Native	NA	SDTF	Bark	Bitter	Stroke	IPA91611
*Vismia guianensis* (Aubl.) Choisy	Hypericaceae	Lacre	Native	Phe, Fla, Tan and Ter	TR	Bark, leaves and roots	Tasteless	Arterial hypertension and Renal problems	IPA91717
*Ximenia americana* linn	Olacaceae	Ameixa	Native	Alk, Phe, Fla, Tan and Ter	SDTF	Bark and leaves	Bitter	Injury, Throat problems, Stomach problems, Inflammation in general and Gynecological problem	Sterile material
*Xylopia frutescens* aubl	Annonaceae	Imbira Vermelha	Native	Alk, Phe, Fla, Tan and Ter	TR	Seeds	Spicy	Pain in general	Sterile material
*Ziziphus joazeiro* Mart	Rhamnaceae	Juazeiro	Native	NA	TR	Bark	Bitter	Expectorant, Toothache, Flu and colds, Airway inflammation and Cough	Sterile material
					SDTF	Bark, leaves and roots	Bitter	Expectorant, Acariasis and other infestations and Cough	IPA91676

In the fifth column are the classes of compounds attributed to the cited plants, namely Alkaloids (ALK), Phenols (PHE), Flavonoids (FLA), Tannins (TAN) and Terpenes (TER). In the sixth column, SDTF indicates that the species in question was obtained in the Seasonally Dry Tropical Forest, while TR indicates that the species was obtained in the Tropical Rainforest. Cells containing NA mean that this information does not apply to the analyses or that these data were not obtained

### Data analysis

The indication of species equivalent and redundant between and for the SDTF and TR areas was performed through a similarity analysis (Jaccard). A binary matrix containing the information obtained from the informants was created, with plant species as objects and diseases as descriptors. Whenever a species was used for a given disease, the cell was filled with a value of 1. When a species was not used for a given disease, the cell in question was zero. The analyses did not include doubles of the same species.

In cases where the same species was mentioned in both regions, it entered the matrix as two distinct entities (Plant A—TR and Plant A—SDTF). From the binary matrix, the Jaccard similarity matrix was constructed. Doubles of species with more than 50% use overlap were considered 'redundant' (when dealing with plants from the same region) and 'equivalent' (when dealing with plants from different regions). To determine in which categories of flavor the plant species would be categorized, each plant in each region was classified according to the flavor and most cited. The flavor and level of availability considered for each plant are shown in Table 3.

For the analysis of the influence of taste and chemical composition of the establishment of utilitarian equivalence and utilitarian redundancy among plant species, the odds ratio (OR) test was used, which is more appropriate to analyze small values once that the number of equivalent pairs was much smaller than the number of pairs that were not equivalent. This test verified: (1) if pairs formed by plants containing a given taste are more likely to be redundant; (2) if pairs formed by plants containing a given class of chemical compounds are more likely to be redundant; (3) if pairs formed by plants containing a given taste are more likely to be equivalent; (4) if pairs formed by plants containing a given class of chemical compound are more likely to be equivalent. These tests were performed for terpenes, alkaloids, phenolic compounds, tannins, and flavonoids.

The *p* value for each test was calculated by testing the null hypothesis of independence between the variables. The odds ratio calculations were performed by the odds ratio function of the fmsb package of the statistical program R, version 3.2.2 (The R Foundation for Statistical Computing). For the processed tests, *p* < 0.05 was allowed.

## Results

Gathering the selected data, we had 27 diseases and 64 plant species, with seven of the cited species occurring in both SDTF and TR. However, only the 48 plants identified up to the species level and with phytochemical studies were included in the analyses.

### .

We observed that pairs with the same taste are 1.46 times more likely to be redundant than different taste pairs (OR = 1.46, IC = 0.99–2.14, *p* = 0.05). Several reports and citations recorded in the interviews on the taste of the species also allow inferring that in the communities studied, the taste is used as a trail of therapeutic efficacy. When asked about the taste of *Mesosphaerum pectinatum* (L.) Kuntze, a species used as an analgesic, a 66-year-old informant living in the community of Dom Helder Câmara states that "for pain, the bitter the better … nothing that is sweet suits." On another occasion, also in this community, a 61-year-old informant emphasized that "For the flu, the remedy has to be bitter … can not suck sugarcane or eat couscous," referring to the bitter taste of the species *Solanum paniculatum* L.

Based on the informants' comments, including those mentioned above, there also seems to be a consensus that the bitter taste signals medicinal value. The relationship between the unpleasant taste, usually bitter, and perceived therapeutic efficacy have also been registered, as follows: "the healing remedy is bad to take! not good, no! "- Informant from Dom Helder Câmara community, 75 years.

In another community, people have shown caution regarding the use of bitter plants. This is clear in practically all the interviews, as one can note in the following comment: "if you drink too much of this tea it is intoxicating because it is very bitter"—Informant of the Batinga community, 27 years old. Something also common in the informants' speech was the relationship between plant bitterness and abortifacient properties, and it is common to hear comments such as "pregnant women cannot take mororó (*Bauhinia acuruana* Moric.) because it is bitter"—Informant from the Batinga community, 41 years old.

Concerning the chemical compounds, there was no positive correlation between the pairs that had a given class of compound and the establishment of redundancy, as can be observed in the results for alkaloids (OR = 1.07, IC = 0.51–2.24, *p* = 0.8659), tannins (OR = 1.3, IC = 0.48–3.50, *p* = 0.606), flavonoids (OR = 2.56, IC = 0.60–10.90, *p* = 0.1891) and terpenes (OR = 1.56, IC = 0.53–4.53, *p* = 0.4144). Furthermore, contrary to what we expected, there is less chance of pairs that share phenols being redundant than pairs that do not (OR = 0.27, IC = 0.12–0.61, *p* = 0.0008319).

The analysis showed that in the scenario investigated there is no relation between the taste attributed to a plant and the Utilitarian Equivalence, (OR = 1.08, IC = 0.71–1.66, *p* = 0.70). The analyses also indicate that, in general, the classes of compounds studied do not affect Utilitarian Equivalence, as shown by the results for the flavonoids (OR = 1.84, IC = 0.55–6.22, *p* = 0.318), Phenols (OR = 0.72, IC = 0.26–1.96, *p* = 0.5139), tannins (OR = 2.21, IC = 0.65–7.63; *p* = 0.193), and terpenes (OR = 1.08, IC = 0.37–3.21, *p* = 0.8849). Regarding alkaloids, the figures associated with the influence on equivalence were not significant as well but were very close to that (OR = 2.21, IC = 0.96–5.12; *p* = 0.057). Thus, in the specific case of alkaloids, there may be a relationship.

## Discussion

### There is more chance of occurrence of utilitarian redundancy among species with similar tastes: the same does not occur with utilitarian equivalence

The fact that taste does not contribute to the establishment of utilitarian equivalence, but favors the establishment of utilitarian redundancy, may indicate that the cultural factor on the perception of taste predominates over any biological factor, intrinsic to any person in any social group. Pieroni and Torry [105] had already documented that different ethnic groups describe the same plants with different flavors, and that flavor was linked to medicinal use, particularly in traditional groups. What we saw in our work is that the same flavor perception in different places (regardless of whether it is recognized in different plants) does not determine the same medicinal use. That is, the perception that something is good for x disease because it is "bitter" or "astringent" is influenced by the cultural context in which they are inserted. In another location, a person may perceive the same taste, but not attribute it to an x disease. In this sense, our work has a great contribution toward finding the role of flavor in different cultural groups.

Our data on the relationship of taste with redundancy seem to support the findings of other studies elsewhere in the world. As previously mentioned, it seems that in different places around the world, tastes are used to identify different medicinal functions. For example, bitter­tasting plants are indicated for the treatment of inflammation in some places [13] and for gastrointestinal disorders in other places [12].

Based on the comments made by informants from the communities studied in TR and SDTF regions, we can see that the same bitter taste has different interpretations. For example, we found comments related to bitter taste as something toxic and abortive, in SDTF region, while in TR region, people say that that any good medicine has to be bitter, such as the statements made by two informants of Ass. D. Helder Câmara, "healing remedy is bad to take! not good", and "for pain, the bitter the better … nothing that is sweet suits." These citations probably correspond to behaviors that, based on previous positive experiences involving the use of bitter-tasting medicinal plants, may guide future episodes of plant selection for these same therapeutic purposes. This is perhaps the main selection mechanism involving taste.

A point to be discussed about our results is the influence of environmental aspects of each region on the production of bioactive compounds and, consequently, on the taste used as a clue. Studies have shown that biochemical routes benefit certain compounds in SDTF and others in TR. In the case of the TR, there are indications that environmental conditions favor biochemical routes related to alkaloid production [106] whereas in SDTF biochemical routes seem to benefit the production of phenolic compounds [106]. Therefore, learning events from the experimentation of floras from distinct environments may make it possible that the compound, and consequently, the taste that treats a particular disease in the SDTF to be different from that used in the TR.

The taste proved to be important for the selection of medicinal plants. People in distinct places consider the taste when choosing their plants. However, depending on the cultural and environmental issues of each local population, the same taste can be attributed to different functions. This shows that there is indeed a trail of chemical efficacy [1] that gradually makes people relate certain tastes to certain diseases in each cultural and environmental context in which they operate [1].

Although it is clear that taste serves as a trail for therapeutic efficacy, the findings that a taste corresponds to a therapeutic activity (through trial and error) are closely related to the chemical repertoire of the local flora. In the case of, for example, a much larger spectrum of phenolic compounds over others, local knowledge on taste versus therapeutic activity may reflect this particularity. In other words, under a parsimonious look, the relationship between taste and medicinal use will always depend on a chemical repertoire available, [2].

### In general, it is not the classes of compounds that determine the utilitarian redundancy and utilitarian equivalence

The mere presence of a class of compounds is not decisive for the occurrence of utilitarian redundancy and equivalence. But in the specific case of alkaloids, it might be relatable that pairs of species with the same class of chemical compounds are equivalents, because of the confidence interval that is almost 1. The result for this class of compound is quite different from those observed for the other ones. The data indicate a tendency for alkaloids to be present twice as expressively in equivalent plant pairs.

Based on the assumption that a large part of the secondary metabolites are intended to inhibit herbivory [107], it is believed that classes of low molecular weight compounds, such as alkaloids, can present bioactivity, even though they occur at low concentrations. In contrast, high molecular weight secondary metabolites, such as tannins, require high concentrations for biological activity to be satisfactory [107, 108]. As the present study used information from studies that aimed at determining the presence or absence of classes of compounds, the data presented here do not capture this flexible and dependent nature of plant concentrations. These are necessary to allow the existence of a pharmacological activity capable of sustaining the therapeutic use of a given plant species in a local community. Conversely, we can infer that the likely positive relation between Utilitarian Equivalence and the double presence of alkaloids may be related to the high bioactivity of these compounds, albeit in low concentrations.

As already discussed, an important aspect of the results is the influence of abiotic conditions on the production of secondary metabolites [109]. The present study reflects the use of plants occurring in semi-arid and humid areas. It is likely that the difference in environmental conditions is influencing the production of medicinal value compounds and, considering that there were species in both SDTF and TR with different uses, these may not meet the same therapeutic demands due to environmental influences. In this sense, there is evidence that factors such as seasonality, temperature, water availability, ultraviolet radiation; nutrient availability, and altitude may alter the amount and types of plant chemical constituents [109]. For example, a positive relationship between tannin production and environmental conditions has been found in the semi-arid region of Northeast Brazil [110–112]. Thus, from the ecological point of view, SDTF plants would have more conditions to produce tannins than TR plants.

Another point that deserves discussing is the possibility that different classes of compounds may promote similar biological activities. Thus, it may be that two plants are used to treat a certain disease, but distinct compounds, not shared between plants, are responsible for the combined therapeutic activity. Following this reasoning, if species treat general illnesses, then pairs of equivalent plants would not necessarily need to have the same class of compound because other classes of compounds act similarly to cure this disease range.

In addition to these elements of ecological influence, many local communities, inhabiting mainly dry forests, have developed strategies for medicinal use that are different from those used by communities that inhabit humid forests [113]. Due to the semi-arid regime and the scarcity of leaves and fruits during most of the year, the behavior of prioritizing the use of tree barks to the detriment of other parts was developed [113]. Although they share chemical compounds, bark and leaves often have distinct concentrations of the same compounds [114]. As a result, tree species that have a low concentration of a given bioactive compound in the bark and a higher concentration in the leaves may not present satisfactory results for the treatment of certain diseases depending on the way they are used by SDTF populations. Thus, double presence alone would not be enough to guarantee Utilitarian Equivalence, especially for high molecular weight compounds such as tannins [107, 108].

It is important to point out that other factors can act together with bioactive compounds and/or with organoleptic characteristics, influencing the healing process and the behavior of the use of medicinal resources [115]. According to Moerman and Jonas [116], there is a meaning response effect, whose healing effect is due more to psychological circumstances than to the existence of bioactive compounds in a resource [116]. When the cure from a given treatment is taken for granted, an immune response occurs, expanding the efforts and thus the energy used by the human organism to treat a disease [117]. Following this perspective, the use of bitter-tasting plants in a cultural context that considers this organoleptic characteristic to be effective in treating diseases can favor the success of the treatment. In this scenario, the habit of using medicinal plants with a bitter taste could be considered an adaptive behavior and thus would tend to settle in the local culture. Therefore, the meaning response can partly explain the existence of usage patterns of plants.

### Limitations

From a methodological viewpoint, this study has some limitations. The first issue concerns collection of taste data. Although the organoleptic property examined in this study was the taste, some people may mix taste and smell into a somehow blurred category. In this sense, some informants spontaneously mentioned the strong smell of some plants, such as mint. However, the information obtained does not allow us to infer the relevance of these characteristics in the local context and we decided not to explain the role of smell in the studied medical system. We relied only on the answers that the informants mentioned the taste, according to their perception of what is taste. Besides that, we accessed the flavor by resorting to people's memories, not by having them taste the plants at the time of the interview. Given the inherent mnemonic limitations of humans, there is a high probability that some flavor has been forgotten.

The second question is about the chemical classification adopted: tannins, phenolics, alkaloids, terpenes, and flavonoids. Initially, the goal was to divide terpenes into small terpenes and large ones, as far as they have very distinct pharmacological properties. However, we found a very small amount of phytochemical studies for these subclasses of compounds, which made it impossible to perform statistical analyzes. It can also be that the absence of findings concerning chemical classes reflects the small size of your sample and our wide chemical classifications. Thus, as this is a first theoretical approach, we find it reasonable to conduct a study that considers the large classes of compounds.

## Conclusion

Taste is an important clue in the selection of medicinal plants by local populations. However, based in our results this clue operates in a different way depending on the cultural and environmental aspects of each local population. It means that, although populations take taste into account in order to include a medicinal plant in their medical system, different factors cause a taste to be attributed to a particular therapeutic function. Plants with the same taste tend to have the same function within but not between local medical systems.

Our results do not allow us to say that the class of plants chemical compounds influences the selection of medicinal plants for the therapeutic categories. However, the supposed influence of the presence of alkaloids for the establishment of Utilitarian Equivalence make us suspect that the chemical composition of plants can contribute to the establishment of certain patterns of use of plants. At this point, we emphasize that the data on the chemical repertoire of plants were limited to the presence or absence and were obtained from the available scientific literature. Consequently, it was not possible to know the actual tissue concentration of the compounds in the plants used in situ, which we considered a limitation of our results.

The empirical data of the present study corroborate the main prediction of the Utilitarian Equivalence Model that plants of distinct medical systems tend to share certain traits in common.

## Data Availability

All data generated or analyzed during this study are included in this published article.

## Abbreviations

URM: Utilitarian Redundancy Model
UEM: Utilitarian Equivalence Model
SDTF: Seasonally Dry Tropical Forest
TR: Tropical Rainforest
